# Organisation of cingulum bundle fibres connecting the anterior thalamic nuclei with the rodent anterior cingulate and retrosplenial cortices

**DOI:** 10.1177/2398212820957160

**Published:** 2020-09-09

**Authors:** Emma J. Bubb, Andrew J. D. Nelson, Thomas C. Cozens, John P. Aggleton

**Affiliations:** School of Psychology, Cardiff University, Cardiff, Wales, UK

**Keywords:** Cortico-thalamic, mouse, rat, thalamo-cortical, thalamus

## Abstract

Despite considerable interest in the properties of the cingulum bundle, descriptions of the composition of this major pathway in the rodent brain have not kept pace with advances in tract tracing. Using complementary approaches in rats and mice, this study examined the dense, reciprocal connections the anterior thalamic nuclei have with the cingulate and retrosplenial cortices, connections thought to be major contributors to the rodent cingulum bundle. The rat data came from a mixture of fluorescent and viral tracers, some injected directly into the bundle. The mouse data were collated from the Allen Mouse Brain Atlas. The projections from the three major anterior thalamic nuclei occupied much of the external medullary stratum of the cingulum bundle, where they were concentrated in its more medial portions. These anterior thalamic projections formed a rostral-reaching basket of efferents prior to joining the cingulum bundle, with anteromedial efferents taking the most rostral routes, often reaching the genu of the corpus callosum, while anterodorsal efferents took the least rostral route. In contrast, the return cortico-anterior thalamic projections frequently crossed directly through the bundle or briefly joined the internal stratum of the cingulum bundle, often entering the internal capsule before reaching the anterior thalamus. These analyses confirm that anterior thalamic connections comprise an important component of the rodent cingulum bundle, while also demonstrating the very different routes used by thalamo-cortical and cortico-thalamic projections. This information reveals how the composition of the cingulum bundle alters along its length.

## Introduction

The cingulum bundle is one of the most prominent white matter tracts in the mammalian brain. Interest in this tract has markedly increased with the growing realisation of its likely involvement in numerous psychiatric conditions (Bracht et al., 2015; Bubb et al., 2018; Daniels et al., 2013; Shepherd et al., 2012). In the primate brain, the cingulum bundle spans much of the length of the medial cerebrum, from the orbital frontal cortices to near the temporal pole (Mufson and Pandya, 1984; Schmahmann and Pandya, 2006). The tract is, however, highly complex as fibres continually appear to join and leave the length of the bundle (Heilbronner and Haber, 2014; Mufson and Pandya, 1984). While tracing studies in nonhuman primates provide detailed information into the changing composition of this pathway (Bubb et al., 2018; Heilbronner and Haber, 2014; Mufson and Pandya, 1984), less appears to be known about the composition of different parts of the rodent cingulum bundle.

In a pair of landmark studies, Domesick (1969, 1970) concluded that efferents from the anterior thalamic nuclei to the cingulate and retrosplenial cortices form major components of the rat cingulum bundle. Following rostral thalamic electrolytic lesions (Domesick, 1970), degenerating axons from the anterior thalamus were observed streaming forward to form fascicles in the dorsomedial aspect of the caudoputamen (see also Clark and Boggon, 1933). These fascicles then turned dorsally to join the cingulum bundle close to the genu of the corpus callosum or, more posteriorly, crossed the corpus callosum to join the cingulum (Domesick, 1970). Together, these efferents terminate across the anterior cingulate and retrosplenial cortices (Domesick, 1970). In contrast, the dense, reciprocal cortico-thalamic projections appeared to take a different route (Domesick, 1969). Rather than becoming enclosed in the sagittal course of the cingulum, these fibres passed directly through it and the underlying callosal strata to reach the thalamus, often traversing the internal capsule to enter the thalamus from its lateral margin (Domesick, 1969).

Despite the lasting influence of these studies (Domesick’s, 1969, 1970), the lesion-degeneration method has multiple limitations. It is not possible to distinguish degenerating fibres that arise from neurons in the target area from damaged fibres of passage. Furthermore, the surgical methods employed by Domesick (1970) make it difficult to separate the contributions of individual anterior thalamic nuclei or exclude other adjacent nuclei. In the case of reciprocally connected sites, there can be the added challenge of distinguishing efferent from afferent fibres (Brodal, 1981). The degeneration method also lacks sensitivity, leaving the possibility of false negatives, that is, fibres that follow alternate routes may have been overlooked (Brodal, 1981). Although subsequent studies using modern axonal tracers injected into the anterior thalamus (Shibata, 1993a; Van Groen et al., 1999), cingulate cortex (Shibata and Naito, 2005) and retrosplenial cortex (Van Groen and Wyss, 1990, 1992, 2003) broadly support the conclusions of Domesick (1969, 1970), no axonal tracer study appears to have focused specifically on these white matter pathways, which form a key link in Papez circuit (Papez, 1937).

The present study was in two complementary parts. The first used a variety of anterograde and retrograde tracers to examine the contributions to the rat cingulum bundle of the reciprocal anterior thalamic – cortical fibres. The second used publicly available data from the Allen Mouse Brain Atlas (http://mouse.brain-map.org/) to track the same interconnections in the mouse brain. This database involves numerous cases with injections of a fluorescent-tagged virus (AAV-eGFP) that is transported anterogradely.

## Materials and methods

### Anatomical borders and nomenclature

The boundaries and nomenclature for the anterior cingulate cortex and anterior thalamus follow those of Paxinos and Watson (2005). Consequently, Cg1 refers to the dorsal and Cg2 to the ventral subdivision of the anterior cingulate cortex (see also Vogt and Paxinos, 2014). For the rat and mouse retrosplenial cortex, the borders and nomenclature follow those of Van Groen and Wyss (1990, 1992, 2003). For this reason, the region is separated into its dysgranular (Rdg), granular a (Rga) and granular b (Rgb) subdivisions. In addition, the Allen Mouse Brain Atlas distinguishes an agranular retrosplenial cortex (Rag), a lateral extension of the dysgranular subdivision.

## Experiment 1: rats

### Ethics

All experiments were performed in accordance with the UK Animals (Scientific Procedures) Act (1986) and associated guidelines and were approved by the local ethical review committees at Cardiff University.

### Animals

Subjects were 29 male Lister Hooded rats (Envigo, Bicester, United Kingdom). They were housed in groups of two or three under a 12-h light/12-h dark cycle, with Lignocel sawdust bedding (IPS Ltd, London, United Kingdom). Prior to surgery, animals were food restricted to maintain at least 85% of their free-feeding body weight. Following surgery, food and water were available ad libitum.

### Overview of injections

Table 1 summarises the virus and tracer injections. Five animals received injections of different tracers in opposite hemispheres, resulting in a total of 34 injection cases.

**Table 1.** Summary of rat cases with injections involving the anterior thalamic nuclei, anterior cingulate cortex, retrosplenial cortex or cingulum bundle.

Case number	Virus/tracer	Injection site
***Coronal cases***		
*Anterogradely transported virus injections in anterior thalamic nuclei*		
215#1	eGFP	AM (CM, MD)
215#12	eGFP	AM (Re)
218#1R	eGFP	AM (AV, AD)
211#3	eGFP	AM (AV, AD, CM, MD)
211#5	eGFP	AM (AV, AD, CM)
211#21	eGFP	AM (AV, VA, CM, MD)
218#1L	eGFP	AV (AM, VA, LD)
218#3	eGFP	AV/AM (VA, CM)
211#13R	eGFP	AV (AM, VA, CM)
211#14	eGFP	AV (AM, VA, CM)
229#2L	eGFP	AV/AD (AM)
229#2R	eGFP	AV/AD (AM)
211#13L	eGFP	AV/AD/AM (VA, CM, MD, LD)
*Anterogradely transported virus injections in anterior cingulate cortex*		
224#1	eGFP	ACC, including pregenual
224#2	eGFP	ACC, including pregenual
219#2	eGFP	ACC, including pregenual
219#18	eGFP	ACC, postgenual
224#20	eGFP	ACC, postgenual
224#21	eGFP	ACC, postgenual
215#31	eGFP	ACC, postgenual
*Anterogradely transported virus injections in retrosplenial cortex*		
224#29	eGFP	Rgb (Rdg)
224#30	eGFP	Rgb
*Anterogradely transported tracer injections in retrosplenial cortex*		
188#5	BDA	Rgb/Rdg (Rga)
187#9	BDA	Rgb (Rdg, Rga)
*Anterogradely and retrogradely transported tracer injections into cingulum bundle*		
205#6	CTB	Cingulum, anterior
209#16	CTB	Cingulum, anterior
209#17	CTB	Cingulum, anterior
205#5	BDA	Cingulum, anterior
205#2R	CTB	Cingulum, posterior
205#2L	BDA	Cingulum, posterior
205#3	BDA	Cingulum, posterior
209#19	BDA	Cingulum, posterior
***Sagittal cases***		
*Anterogradely and retrogradely transported virus injections in anterior thalamic nuclei*		
213#2L	EIAV	AV/AD/LD
213#2R	EIAV	AV/AD/LD

ACC: anterior cingulate cortex; AD: anterodorsal thalamic nucleus; AM: anteromedial thalamic nucleus; AV: anteroventral thalamic nucleus; BDA: biotinylated dextran amine; CM: central medial nucleus; CTB: cholera toxin subunit B; eGFP: green fluorescent protein-tagged adeno-associated virus, AAV5-CaMKIIa-eGFP; EIAV: Equine infectious anaemia virus; LD: laterodorsal thalamic nucleus; MD: mediodorsal thalamic nucleus; Re: nucleus reuniens; Rdg: retrosplenial cortex, dysgranular region; Rga and Rgb: retrosplenial cortex granular area a and b; VA: ventral anterior thalamic nucleus.

Cases are grouped by injection site. For some cortical sites, multiple injections helped to ensure sufficient spread across the target region. Cases with injections in different hemispheres are indicated by L (left) and R (right). Sites in parenthesis indicate weak involvement from the injection site.

One of the tracers was the anterogradely transported green fluorescent protein-tagged adeno-associated virus AAV5-CaMKIIa-eGFP (‘AAV-eGFP’). Injections of AAV-eGFP (titre: 4.3 × 10^12^ GC/mL, Addgene, Watertown, MA, USA) targeted the anterior thalamic nuclei, the anterior cingulate cortex and retrosplenial cortex. In addition, the tracers biotinylated dextran amine (BDA, 3 kD, Life Technologies Ltd, Paisley, United Kingdom) and conjugated cholera toxin subunit B (CTB, Invitrogen, Carlsbad, CA, USA) were injected into the cingulum bundle itself, above the corpus callosum. The BDA was made up at 10% in sterile, distilled water (pH: 7.4) and CTB was made up at 1% in 0.1-M phosphate-buffered saline (PBS). Both tracers are transported both anterogradely and retrogradely, with a stronger anterograde component for BDA (Veenman et al., 1992) and a stronger retrograde component for CTB (Dederen et al., 1994). Further BDA injections targeted the retrosplenial cortex. A final pair of cases received injections of the anterogradely and retrogradely transported Equine infectious anaemia virus (EIAV, Invitrogen, Renfrewshire, United Kingdom; see Barker et al., 2017) within the anterior thalamic nuclei (Table 1). Typically, individual injection volumes for viruses ranged between 0.4–0.7 and 0.08–0.1 µL for tracers.

### Surgical procedures

All animals weighed between 290 and 380 g at the time of their surgeries. Anaesthesia was induced using a mixture of oxygen and 5% isoflurane. Once unresponsive, the isoflurane level was lowered to 1.5%–2.5% for the remainder of the surgery and animals were placed in a stereotaxic frame (David Kopf Instruments, Tujunga, CA, USA). For injections into the anterior thalamic nuclei and retrosplenial cortex, the incisor bar was set so that the skull was at +5 mm relative to the horizontal plane. For injections into the anterior cingulate cortex and cingulum bundle, the skull was flat relative to the horizontal plane. Animals were administered a subcutaneous injection of the analgesic Metacam (0.06 mL, Boehringer Ingelheim Ltd, Bracknell, United Kingdom), and the analgesic lidocaine (0.1 mL, Xylocaine, AstraZeneca, Luton, United Kingdom) was applied topically above the surgical site. A sagittal incision was made, allowing the scalp to be retracted, followed by a craniotomy over the injection sites.

All virus injections were delivered with a 10-μL Hamilton syringe (Bonaduz, Switzerland) controlled by a microprocessor (World Precision Instruments, Hitchin, United Kingdom) set to a flow rate of 0.1 μL/min, with the needle left in situ for a further 5 min to allow for virus diffusion. All other tracer injections were made iontophoretically using a glass micropipette (18–22-µm tip diameter), using an alternating current (6-s on/off) of 6 µA for 10 min for each injection.

The surgical site was closed using sutures, next the analgesic bupivacaine (Pfizer, Walton Oaks, United Kingdom) and topical antibiotic powder Clindamycin (Pfizer, Walton Oaks, United Kingdom) were applied to the site. Animals were administered a subcutaneous injection of glucose-saline (5 mL) for fluid replacement before being placed in a recovery chamber until they regained consciousness. Animals were monitored carefully postoperatively with food available ad libitum until they appeared fully recovered.

### Histology

Following a post-operative survival time that varied by tracer/virus (BDA and CTB, 1 week; eGFP, 3–6 weeks; EIAV, 5 weeks), animals were administered an intraperitoneal injection of a lethal dose of sodium pentobarbital (2 mL/kg, Euthatal, Marial Animal Health, Harlow, Essex, United Kingdom) and transcardially perfused with 0.1-M PBS, followed by 4% paraformaldehyde (PFA) in 0.1-M PBS. Brains were removed, post-fixed in PFA for 2 h and then placed in 25% sucrose solution for 24 h at room temperature on a stirring plate. Brains were cut into 40-μm sections using a freezing microtome (8000 sledge microtome, Bright Instruments, Luton, United Kingdom) and a series of 1 in 4 sections was collected in PBS. For eGFP injections, enhancement of the fluorescence signal was not necessary and, therefore, no immunohistochemistry was performed. Apart from the EIAV cases, all brains were sectioned coronally. The two EIAV cases were sectioned in the sagittal plane to help reveal the course of the bundle.

For the EIAV injections, sections were mounted onto gelatine subbed slides, covered in X-gal solution (50% X-gal, 1.25% dimethyl sulfoxide, 5% 50-mM potassium ferricyanide, 5% potassium ferrocyanide, 1% octylphenoxypolyethoxyethanol (1% concentration), 0.5% sodium deoxycholate (1% concentration), 2% magnesium chloride, in PBS) and incubated in a water bath at 37°C for 5 h. The X-gal solution was then removed, and sections were washed three times by applying PBS to the slides. Slides were left to dry overnight before being immersed in distilled water for 90 s, counterstained in eosin for 60 s, and immersed in distilled water for a further 60 s.

For BDA injections, sections were first washed three times in tris-buffered saline (TBS). They were then incubated on a stirring plate at room temperature for 2 h with fluorophore (A488) conjugated streptavidin (Thermo Fisher, United Kingdom), at a dilution of 1:200 in TBS with 1% normal goat serum (NGS) and 0.2% Triton X-100. Sections were then washed three times in TBS, twice in Trizma non-saline (TNS).

For CTB injections, sections were first washed three times in PBS followed by once in phosphate-buffered saline with Triton X-100 (PBST). Sections were then transferred into a blocking solution of 5% NGS in PBST and incubated for 90 min. Sections were then moved into the primary antibody solution of rabbit-anti-cholera toxin (Sigma Aldrich, Gillingham, United Kingdom) at a dilution of 1:10,000 in PBST with 1% NGS, and incubated for 24 h. Sections were then washed four times in PBST and moved to a secondary antibody solution of goat-anti-rabbit (DyLight Alexa fluor 594, Vector Laboratories, Peterborough, United Kingdom) at a dilution of 1:200 in PBST with 1% NGS. Sections were incubated for 2 h and then placed in a refrigerator (4°C) overnight before being washed four times in PBST. All incubations were on a stirring plate at room temperature, and all washes were for 10 min unless otherwise stated.

Following immunohistochemistry, sections were mounted onto gelatine subbed glass slides. All slides dried overnight before being immersed in xylene and coverslipped using dibutylphthalate polystyrene xylene (DPX) (Thermo Fisher Scientific, Loughborough, United Kingdom). Injection sites and virus/tracer transport were analysed using a fluorescent Leica DM5000B microscope with a Leica DFC310 FX camera.

## Experiment 2: mice

Cases were selected from the Allen Mouse Brain Atlas (connectivity.brain-map.org). In all cases, the anterogradely transported adeno-associated virus (AAV), tagged with green fluorescent protein (eGFP), was injected into the target region (see connectivity.brain-map.org for further details, including the sensitivity of this method). Following a search for all cases with injections listed as being in either the anterior cingulate cortex, retrosplenial cortex or individual anterior thalamic nuclei, further criteria were applied. First, the target area (e.g. anterior cingulate cortex) was required to receive more than 60% of the injection (as stated in the Atlas). Second, the total injection volume had to be greater than 0.02 mm^3^ and less than 0.30 mm^3^. Any cases that did not show transport of the virus from the injection site were excluded. All mice were adults of more than 10 weeks old. The cases include many genetically modified animals, so that comparisons involved wild-type mice whenever possible.

These criteria reduced the number of cases with acceptable injections in the anterior cingulate cortex from a total of 102 to 57 (26 female). Likewise, retrosplenial cortex cases reduced from 111 to 47 (17 female), anteromedial thalamic nucleus cases from 12 to 4 (3 female) and anteroventral thalamic nucleus cases from 12 to 5 (2 female). Each case was then examined, and representative examples that typified the patterns of projections from each of regions of interest received further analysis. These were typically those in which the injections were best restricted to the target area. Care was taken to ensure that any consistent pathway variations from within an injection area were retained in the representative cases.

Three representative examples were selected from the anterior cingulate cortex, which together encompassed both dorsal (Cg1) and ventral (Cg2) subdivisions and most of the anteroposterior length of the region (Table 2). Three representative examples were selected from the retrosplenial cortex, with injections that together involved both the granular and dysgranular subdivisions and covered the anteroposterior extent of the region. One example was selected for the anteromedial thalamic nucleus, while two anteroventral thalamic nucleus were included. These thalamic cases were those where the injection site was most confined within that nucleus (anteromedial) or where a wild-type equivalent was also present (anteroventral). The anterodorsal thalamic nucleus could not be examined separately as no virus injection met the designated criteria.

**Table 2.** Summary of illustrated Allen Mouse Brain Atlas cases with tracer injections in the anterior cingulate cortex, retrosplenial cortex, anteromedial thalamic nucleus or anteroventral thalamic nucleus.

Experiment number	Gender, age, genetic status and strain	Injection volume (mm^3^)	Sites injected (% of injection size)
**286482701**	Female, 11 weeks, Rbp4-Cre_KL100	0.229	ACC: Cg1 (36%), Cg2 (63%)
**299829892**	Male, 11 weeks, Syt6-Cre_KI148	0.123	ACC: Cg1 (25%), Cg2 (74%)
**126190033**	Female, 11 weeks, Syt6-Cre_KI148	0.077	ACC: Cg2 (97%), fibre tracts (3%)
**100140949**	Male, 10 weeks, C57BL/6J	0.185	RSC: Rgb/Rga (73%), Rdg (27%)
**482581199**	Female, 11 weeks, Rbp4-Cre KL100	0.205	RSC: Rdg (58%), Rgb/Rga (32%), Post (5%), VISp (2%), APr (2%)
**112424813**	Male, 11 weeks, C57BL/6J	0.030	RSC: Rdg (100%)
**114427219**	Male, 11 weeks, C57BL/6J	0.170	AV (77%), AD (20%)
**286553311**	Female, 11 weeks, Gpr26-Cre_KO250	0.040	AV (99%)
**573035760**	Male, 11 weeks, Prkcd-GluCla-CFP-IRES-Cre	0.052	AM (70%), IAD (16%), IAM (7%), MD (2%), PT (4%)

ACC: anterior cingulate cortex, dorsal (Cg1) and ventral (Cg2) subdivisions; AM: anteromedial thalamic nucleus; Apr: area prostriata; AV: anteroventral thalamic nucleus; IAD: interanterodorsal nucleus; IAM: interanteromedial nucleus; MD: mediodorsal thalamic nucleus; Post: postsubiculum; PT: parataenial thalamic nucleus; RSC: retrosplenial cortex, granular a (Rga), b (Rgb) and dysgranular (Rdg) subdivisions; VISp: primary visual area.

Abbreviations for sites included in the tracer injections (from the Allen Mouse Brain Atlas).

All cases, which were sectioned on the coronal plane, were examined using the Allen Institute high-resolution image viewer. Representative coronal images were taken from sections showing the fibre trajectories of interest. Additional sagittal images, taken from whole brain reconstructions of each case, were created using serial two-photon tomography (see http://mouse.brain-map.org/).

## Results

### Experiment 1: rats

#### Efferent projections from the anterior thalamic nuclei to cingulate cortex

##### Anteromedial thalamic nucleus

**Figure 1.**
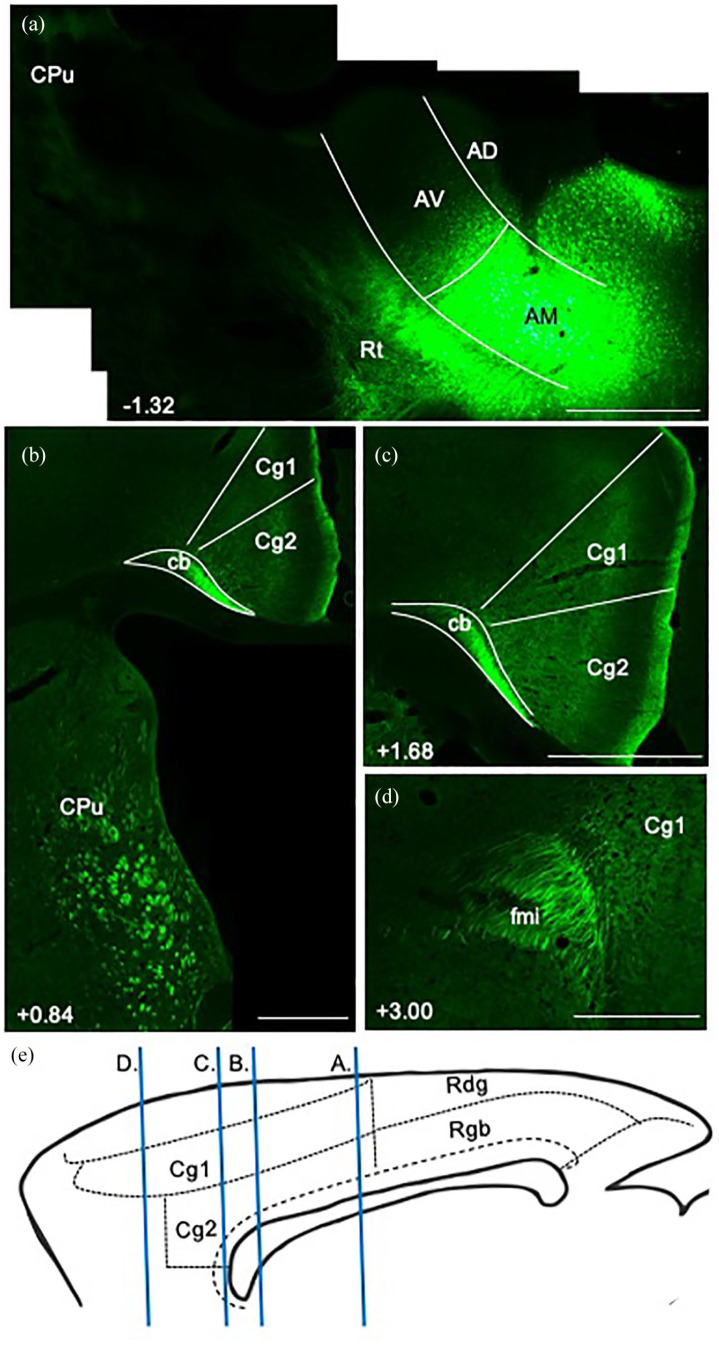
Photomicrographs showing the trajectory of fibres from the anteromedial thalamic nucleus to the cingulate cortex in the rat. Coronal images from eGFP virus injection case 215#1 (from caudal to rostral). Numbers show anteroposterior distance (mm) from bregma. (a) The injection site is centred in the anteromedial thalamic nucleus (AM). Note that much of the label in the reticular nucleus likely reflects anterograde transport involving collaterals to this thalamic area. At this level, there is an absence of labelled fibres in the caudoputamen (CPu). (b) Fibre bundles travelling rostralward through the medial caudoputamen, and fibres travelling caudalward in the medial Cb. (c) Terminal label in the anterior cingulate cortex (Cg1 and Cg2), along with dense fibre labelling in the external medullary stratum of the more medial cingulum bundle (Cb). (d) Fibres wrapping around the genu of the corpus callosum in the forceps minor (fmi), alongside terminal label in the anterior cingulate cortex (Cg1). (e) Sagittal schematic showing the AP levels of images (a)–(d). AD: anterodorsal thalamic nucleus; AV: anteroventral thalamic nucleus; Rdg: dysgranular retrosplenial cortex; Rgb: granular b retrosplenial cortex; Rt: reticular thalamic nucleus. Scale bars 1 mm.

In three cases (215#1, 215#12, 218#1R), anterogradely transported eGFP virus injections were centred in and largely limited to the anteromedial (AM) nucleus of the thalamus (Figure 1(a), case 215#1). In all three cases, a great many fibres left the thalamus via the anterior thalamic radiation (also called the anterior thalamic peduncle) and entered the anterior limb of the internal capsule. Travelling rostralward and dorsalward towards the corpus callosum, multiple discrete fibre fascicles crossed through the medial aspect of the caudoputamen (Figure 1(c)).

From the level of the anterior commissure forward, fibres pierced through the body of the corpus callosum along its anteroposterior axis to join the cingulum from its lateral side. In addition, many fibre fascicles extended beyond the anterior limit of the caudoputamen, turning dorsalward and then caudalward to wrap around the genu of the corpus callosum and join the cingulum (Figure 1(d)). Here, they aggregated in the medial aspect of the external medullary stratum of the cingulum (Figure 1(b)), following its sagittal course caudalward to terminate in layers 1 and 4–6 of the dorsal (Cg1) and ventral (Cg2) anterior cingulate cortex (Figure 1(b)). A light projection reached the retrosplenial cortex, primarily terminating in layer 1 of Rgb. The same fibre pathways were again evident in the three cases with less confined virus injections centred in the anteromedial nucleus (211#3, 211#5, 211#21, see Table 1), despite the injections appearing to reach adjacent parts of the anteroventral (AV) and/or anterodorsal (AD) nuclei.

**Figure 2.**
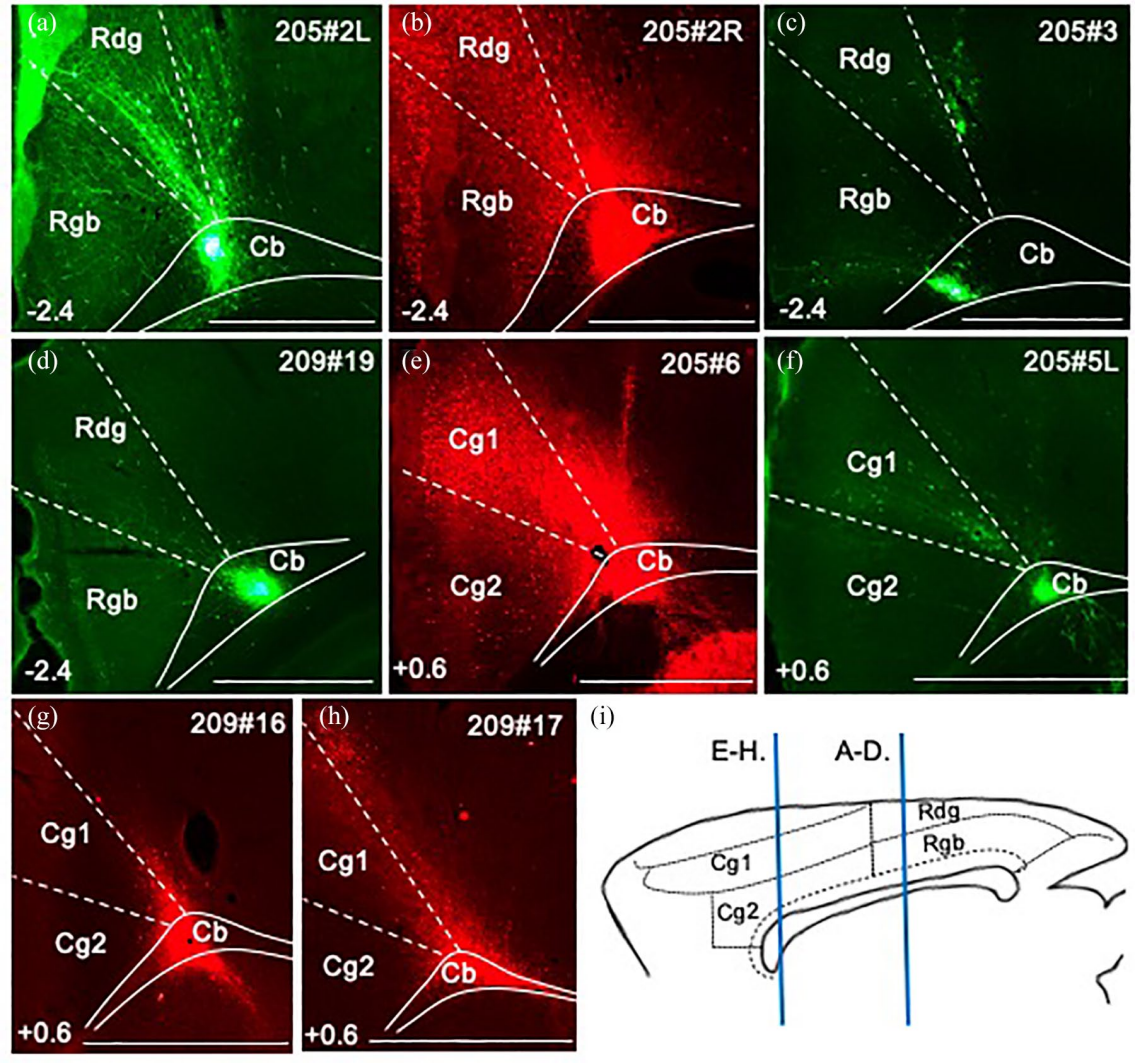
Photomicrographs showing anterogradely and retrogradely transported tracer injections into the cingulum bundle. The coronal green images illustrate biotinylated dextran amine (BDA) injections (a, c, d and f), while the red images show cholera toxin subunit B (CTB) injections (b, e, g and h). The top right number is the case ID. Bottom left numbers show anteroposterior distance (mm) from bregma. (i) Sagittal schematic showing the AP levels of images (a)–(d) and (e)–(h). Cg1: anterior cingulate cortex, dorsal part; Cg2: anterior cingulate cortex, ventral part; Rdg: retrosplenial cortex, dysgranular area; Rgb: retrosplenial cortex, granular area b. Scale bars 1 mm.

**Figure 3.**
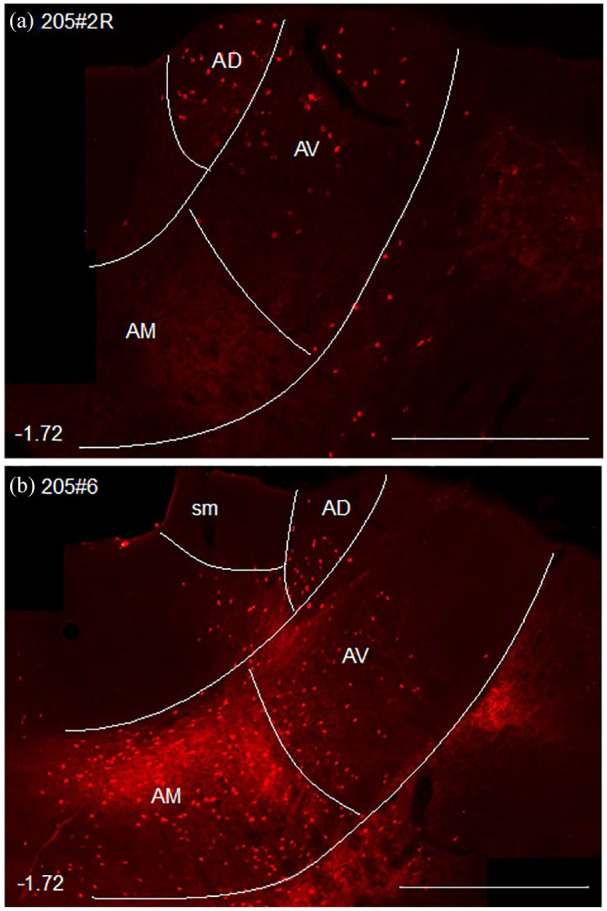
Photomicrographs showing anterogradely and retrogradely transported label in the anterior thalamic nuclei following tracer injections at different anterior–posterior locations in the cingulum bundle (see Figure 2). (a) Label from an injection below retrosplenial cortex (Figure 2(b)). Retrograde label in the anterodorsal (AD) and anteroventral (AV) thalamic nuclei, contrasting with little or none in the anteromedial (AM) nucleus. (b) Anterograde and retrograde label from an injection below the anterior cingulate cortex (see Figure 2(e)). Considerable label in AM (anterograde and retrograde), with appreciably lighter label in AV and AD (the latter only retrograde). Bottom left numbers show anteroposterior distance (mm) from bregma. sm: stria medullaris. Scale bars 1 mm.

In all four cases with tracer injections within the cingulum bundle below the anterior cingulate cortex (Figure 2(e)–(h), 205#5L (BDA), 205#6, 209#16, 209#17 (CTB)), retrograde label was observed in the anteromedial nucleus, consistent with the presence of projections from this thalamic nucleus at this level of the bundle (Figure 3(b)). In contrast, only sparse retrogradely labelled cells were observed in the anteromedial nucleus following tracer injections into the cingulum bundle underneath the retrosplenial cortex (Figure 2(a)–(d), 205#2R (CTB), 205#2L, 205#3 and 209#19 (BDA), Figure 3(a)). Together, these cases indicate that most fibres from the anteromedial nucleus contributing to the cingulum bundle terminate in the anterior cingulate cortex.

##### Anteroventral thalamic nucleus

**Figure 4.**
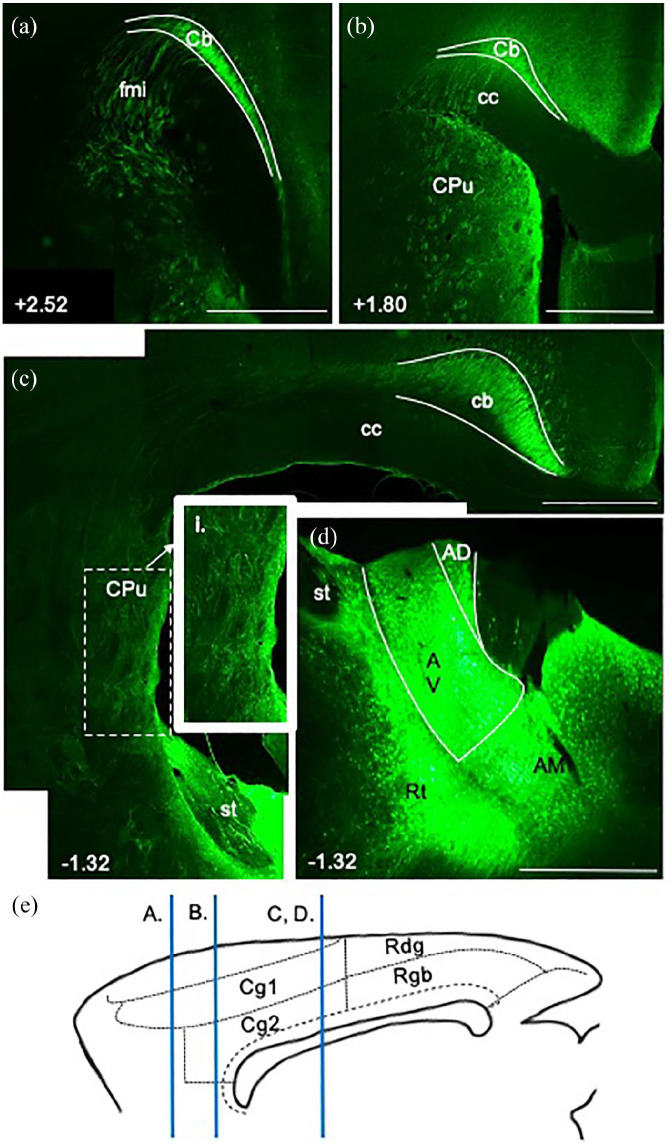
Photomicrographs showing the trajectory of fibres principally from the anteroventral thalamic nucleus to the cingulate cortex in the rat. Coronal images from eGFP virus injection case 229#2L, arranged rostral to caudal (a–d). Numbers show the anteroposterior distance (mm) from bregma. (a) Fibres wrapping around the genu of the corpus callosum in the forceps minor (fmi). (b) Fibres crossing the caudoputamen (CPu) and corpus callosum (cc) to join the cingulum bundle (Cb). (c) Fibres leaving the thalamus laterally to travel dorsalward through the CPu to join the bundle. Fibres occupy the external medullary stratum of the bundle. (i) Inset from (c) showing fibres crossing CPu at higher magnification. (d) Injection site centred in anteroventral (AV) thalamic nucleus. (e) Sagittal schematic showing the AP levels of images (a)–(d). Note that some label in the reticular nucleus (Rt) likely reflects anterograde transport to this thalamic area. AD: anterodorsal thalamic nucleus; AM: anteromedial thalamic nucleus; Cg1, Cg2: anterior cingulate cortex subregions; Rdg: dysgranular retrosplenial cortex; Rgb: granular b retrosplenial cortex; Rt: terminalis. Scale bars 1 mm.

In six cases, anterogradely transported eGFP virus injections were centred in the anteroventral nucleus with varying involvement of other anterior thalamic nuclei (218#1L, 218#3, 211#13, 211#14, 229#2L, 229#2R). Many labelled fibres followed the same initial trajectory as the anteromedial nucleus projections, as detailed in the previous section. This rostral pattern, involving the anterior thalamic radiation, was still present in those cases with the least involvement of the anteromedial nucleus (Figure 4(a), (b) and (d), 229#2L, 218#1L). Furthermore, all tracer injections in the cingulum bundle level adjacent to the anterior cingulate cortex (Figure 2, 205#5L (BDA), 205#6, 209#16 and 209#17 (CTB)) resulted in some retrograde label in the anteroventral nucleus, again consistent with the presence of efferents from this nucleus at this level of the fibre pathway.

In addition, all eGFP virus injections involving the anteroventral nucleus also revealed fibres following more direct routes to the cortex not seen after anteromedial nucleus injections (Figure 4(b) and (c)). Of those fibres leaving the thalamus by the anterior thalamic radiation, many made a sharp dorsalward turn towards the corpus callosum. Consequently, fibres crossed the caudoputamen and entered the cingulum bundle under the entire anteroposterior length of the anterior cingulate cortex. An additional population of fibres left the thalamus laterally, travelling dorsalward around the lateral ventricle (Figure 4(c)) to cross directly into the cingulum bundle under the retrosplenial cortex. In the cingulum bundle, fibres from these injections occupied both the medial and lateral aspect of the external medullary stratum of the cingulum (Figure 4(c)). In comparison to the injections located in the anteromedial nucleus, the cases centred in the anteroventral nucleus resulted in more terminal label in retrosplenial cortex, most evident in layers 1 and 4 of the granular subdivisions.

Given evidence that projections from the anterodorsal thalamic nucleus (next section) and from the laterodorsal (LD) thalamic nucleus (not described here) also often follow a direct, lateral route to the cortex, it is notable that some anteroventral nucleus injections did not appear to involve either of these nuclei (218#1 and 211#14) yet produced this same pattern of fibre labelling. Furthermore, all tracer injections in the cingulum bundle immediately under the retrosplenial cortex (Figure 2(a)–(d), 205#2R (CTB), 205#2L, 205#3 and 209#19 (BDA), Figure 3(a)) resulted in retrograde label in the anteroventral nucleus, again consistent with the presence of projections from this nucleus to this level of the bundle.

##### Anterodorsal thalamic nucleus

Three eGFP injection cases involved the anterodorsal thalamic nucleus (211#13, 229#2L and 229#2R) resulting in labelled fibres following both the rostralward (like anteromedial) and more direct (like anteroventral) trajectories described thus far. However, the involvement of the anteroventral nucleus at the injection site in these same cases precludes the separation of those fibres originating exclusively from the anterodorsal thalamic nucleus, based on anterograde transport.

Only light retrograde label was observed in the anterodorsal nucleus from any of the four tracer injections in the cingulum bundle immediately under the anterior cingulate cortex (Figure 2(e)–(h), 205#5L (BDA), 205#6, 209#16 and 209#17 (CTB), Figure 3(b)). Together, these cases indicate that most projections from the anterodorsal nucleus do not join the cingulum bundle anterior to this level, that is, few efferent fibres from the anterodorsal nucleus follow the rostralward route to the cortex described previously. Further caudal in the cingulum bundle, tracer injections under the rostral retrosplenial cortex (Figure 2(a)–(d), 205#2L (BDA), 205#3 (BDA), 205#2R (CTB), 209#19 (BDA), Figure 3(a)) resulted in relatively more retrograde label in the anterodorsal nucleus. This pattern suggests that most projections from the anterodorsal nucleus take the more direct route to the cortex, that is, often joining the cingulum bundle at anteroposterior levels similar to the injection site.

##### Cases cut in the sagittal plane

**Figure 5.**
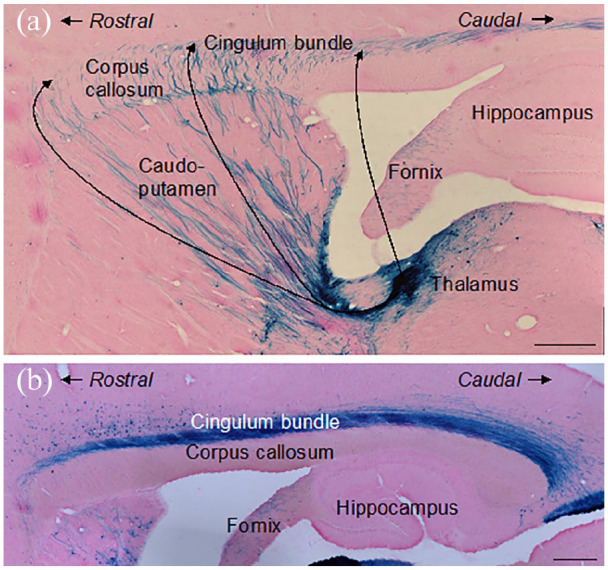
Sagittal photomicrographs showing the trajectory of fibres linking the anterior thalamic nuclei to the cingulum bundle in the rat. Sagittal images from an EIAV injection in the anterior thalamic nuclei, case 213#2. (a) Injection site in anterior thalamic nuclei. Fibres cross the caudoputamen and corpus callosum to join the cingulum along its rostrocaudal length. (b) Fibres fascicles spread out dorsally above the caudal parts of the cingulum bundle. Scale bars 1 mm.

In two cases, EIAV injections were centred in the anterior thalamic nuclei, involving more than one nucleus. These cases were cut in the sagittal plane to reveal more of the length of the cingulum bundle. (Note that the EIAV virus predominantly travels anterogradely but may also travel retrogradely (Barker et al., 2017)). Discrete fibre fascicles were seen crossing the caudoputamen and corpus callosum to join the cingulum along its rostrocaudal axis (Figure 5(a)) as described in the preceding sections. Other fibres turned lateral close to the level of the anterior thalamic nuclei prior to reaching the cingulum bundle more directly. These multiple routes are consistent with the extent of the injection site, which involved both the anteromedial and anteroventral thalamic nuclei. These same cases also revealed that the dorsal cingulum is not tightly bound to the corpus callosum, rather the fibre fascicles become increasingly diffuse above more posterior parts of the tract, that is, above the splenium (Figure 5(b)).

#### Efferent projections from cingulate cortices to the anterior thalamic nuclei

##### Anterior cingulate cortex

**Figure 6.**
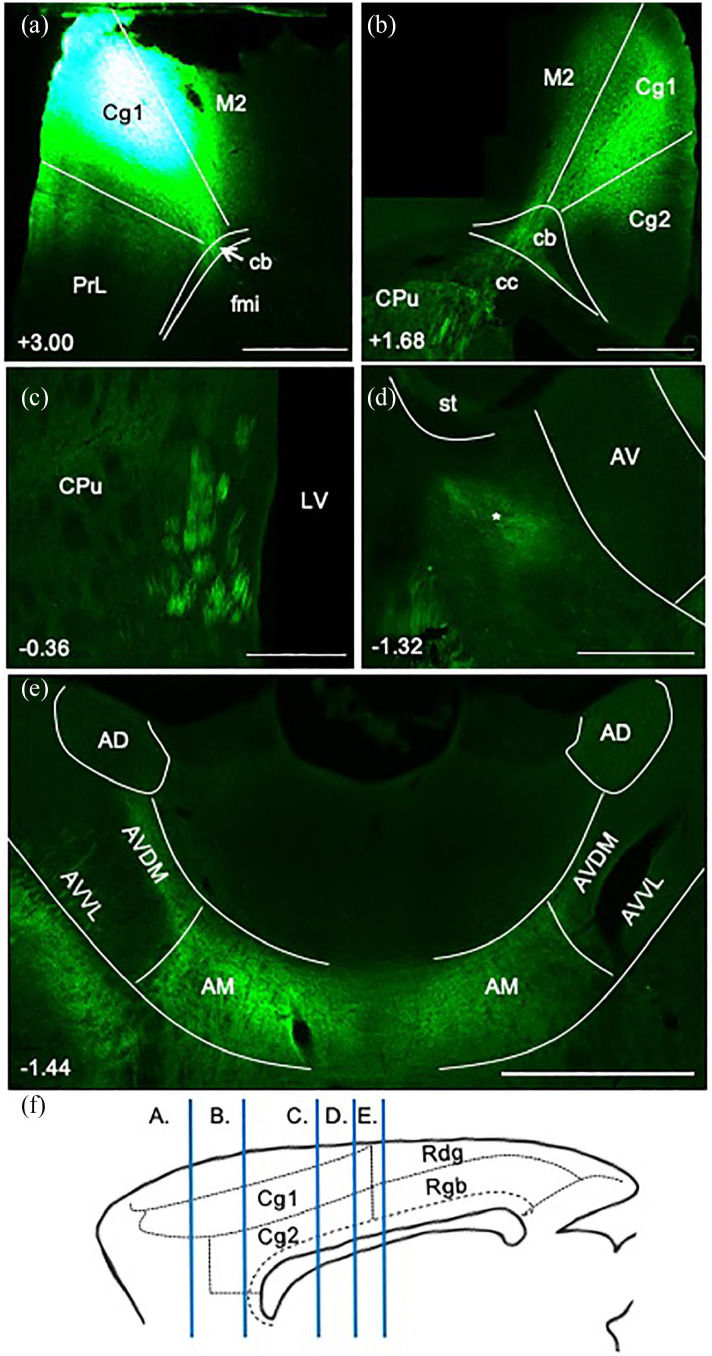
Photomicrographs showing the trajectory of fibres from the anterior cingulate cortex to the anterior thalamic nuclei in the rat. Coronal images from eGFP virus injections (a) in case 224#1 and (b–e) case 215#31, from rostral to caudal levels. Numbers show anteroposterior distance (mm) from bregma. (a) Injection site in pregenual anterior cingulate cortex (Cg1). Fibres entering the cingulum bundle (Cb) to follow its caudalward course. (b) Injection site in postgenual dorsal anterior cingulate cortex (Cg1). Fibres crossing Cb and corpus callosum (cc) to enter the caudoputamen (CPu). (c) Fibres travelling caudalward in the medial CPu. (d) Fibres turning medially (*) to enter the anterior thalamus. (e) Terminal label in the anteromedial (AM) and dorsomedial anteroventral thalamic nuclei (AVDM). Some fibres cross the midline to terminate in same nuclei in other hemisphere, from a unilateral injection. (f) Sagittal schematic showing the AP levels of images (a)–(e). AD: anterodorsal thalamic nucleus; Cg2: ventral anterior cingulate cortex; fmi: forceps minor of the corpus callosum; LV: lateral ventricle; M2: secondary motor cortex; PrL: prelimbic cortex; Rdg: dysgranular retrosplenial cortex; Rgb: granular b retrosplenial cortex; st: stria terminalis. Scale bars 1 mm.

In three cases, viral injections of eGFP were made in the anterior cingulate cortex that extended to the most rostral limit of this region, encompassing all layers of pregenual Cg1 (224#1 (Figure 6(a)), 224#2 and 219#2). Label from this area travelled caudally in the internal stratum of the cingulum bundle above the body of the corpus callosum (Figure 6(a)), before piercing through the white matter (Figure 6(b)) to aggregate in fascicles travelling caudalward and ventralward in the medial aspect of the caudoputamen (Figure 6(c)). After joining the anterior limb of the internal capsule, fibres turned medial around the stria terminalis (Figure 6(d)) to terminate in the anteromedial nucleus and dorsomedial anteroventral nucleus (AVDM) (Figure 6(e)).

Injections of eGFP in the anterior cingulate cortex (Cg1 and Cg2) at the level of the body of the corpus callosum (219#18, 224#20, 224#21) revealed fibres passing through the cingulum bundle directly from the injection site, without becoming enclosed in the white matter for any length. The fibres then followed the same route as described previously and terminated in the same anterior thalamic nuclei as projections from pregenual anterior cingulate cortex (Figure 6(e)). A case with unilateral injections in the anterior cingulate cortex (215#31) revealed that projections reach the contralateral anterior thalamus by the same route, that is, crossing through the ipsilateral thalamus to terminate lightly in the same nuclei in the contralateral hemisphere (Figure 6(e)) (see Mathiasen et al., 2017).

In all four cases with tracer injections into the cingulum bundle under the anterior cingulate cortex (Figure 2(e)–(h) 205#5L (BDA), 205#6 (Figure 3(b)), 209#16, 209#17 (CTB)), anterograde label was observed in the anteromedial and AVDM nuclei, but not in the anterodorsal nucleus. This pattern indicates that the rostral part of the internal stratum of the cingulum bundle contains efferents to just these anterior thalamic nuclei.

##### Retrosplenial cortex

**Figure 7.**
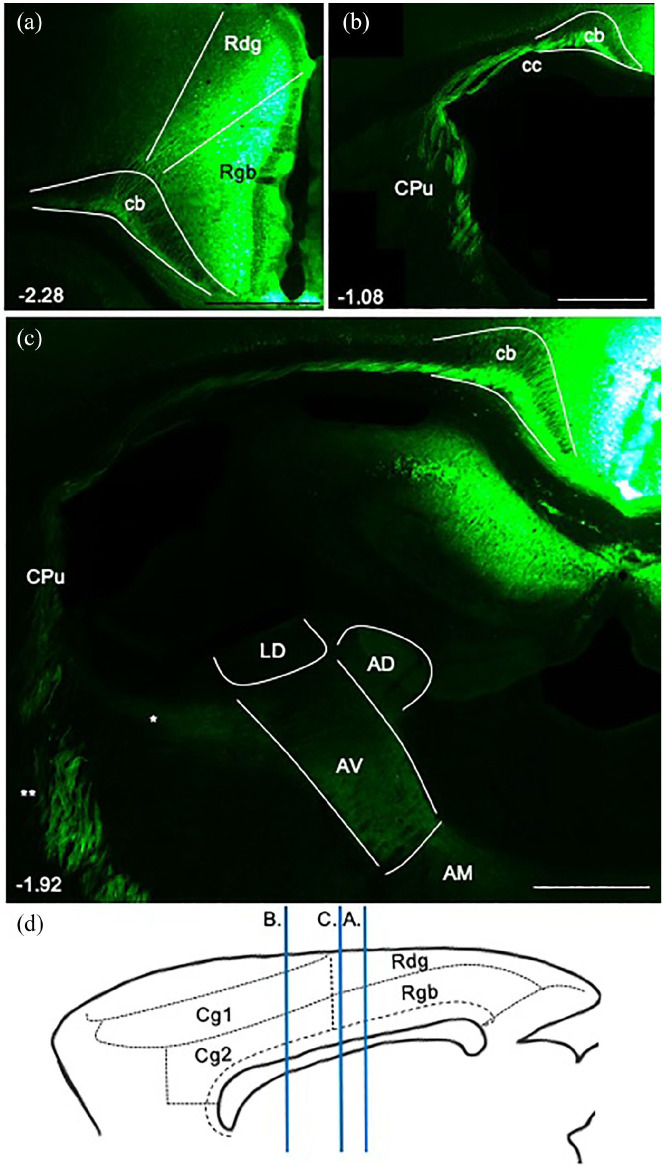
Photomicrographs showing the trajectory of fibres from the retrosplenial cortex to the anterior thalamic nuclei in the rat. Coronal images from unilateral eGFP virus injection case 224#29. (a) Injection site centred in granular retrosplenial area b (Rgb). Fibres enter the cingulum bundle (Cb) to follow its rostralward course. (b) Fibres travelling beyond the rostral limit of the anterior thalamus before crossing the corpus callosum (cc) to enter the caudoputamen (CPu). (c) Fibres crossing the caudoputamen at the level of the anterior thalamus. (*) Fibres turning medially to enter the anterior thalamus, terminal label in the anteroventral (AV) and anterodorsal (AD) thalamic nuclei. Note that label in the anteromedial (AM) thalamic nucleus predominantly comprises fibres that are crossing the midline to terminate in AV and AD in the contralateral hemisphere. (**) Fibres continuing ventrally to reach brainstem targets. (d) Sagittal schematic showing the AP levels of images (a)–(c). CA1: field of the hippocampus; Cg1, Cg2: anterior cingulate subregions; LD: laterodorsal thalamic nucleus; Rdg: dysgranular retrosplenial cortex. Scale bars 1 mm.

In two cases, unilateral eGFP virus injections extended from near the rostral limit of the retrosplenial cortex to just anterior to the splenium (224#29, 224#30), encompassing all layers of Rgb and reaching into Rdg (Figure 7(a)). Fibres joined the internal stratum of the cingulum bundle and travelled rostralward to the level of the anterior thalamus (Figure 7(a)). From here, fascicles cut down through the white matter and caudoputamen, skirting the lateral ventricle to briefly pass through the internal capsule. Taking a sharp medialward turn around the stria terminalis, fibres entered the thalamus from its lateral side (Figure 7(c)). Terminal label was present in the anteroventral and anterodorsal thalamic nuclei (Figure 7(c)), with some fibres seen crossing the midline thalamus to terminate lightly in the same nuclei of the contralateral hemisphere.

A further subset of efferents from these cases entered the internal stratum of the cingulum bundle and projected beyond the rostral limit of the anterior thalamus. These fibres cut through the white matter below the anterior cingulate cortex, that is, at a more anterior level to those fibres described above. These fibres then formed fascicles travelling caudalward and ventralward through the medial caudoputamen (Figure 7(b)). While it is possible that some efferents following this route terminated in the anterior thalamic nuclei, the majority of these fibres entered the posterior limb of the internal capsule (Figure 7(c)). From here, they entered the cerebral peduncle towards targets in the brain stem.

In all four cases with tracer injections into the cingulum bundle below the retrosplenial cortex (Figure 2(a)–(d), 205#2R (CTB), 205#2L, 205#3 and 209#19 (BDA)), anterograde label was observed in the anteroventral thalamic nucleus. This is consistent with efferents from retrosplenial cortex reaching the anteroventral thalamic nucleus via this caudal part of the cingulum bundle. Two of these injections (205#2R (CTB), 209#19 (BDA)) produced light anterograde label in the anterodorsal thalamic nucleus, indicating modest retrosplenial efferents to this nucleus from this level of the cingulum bundle.

## Experiment 2: mice

Both male and female mice were examined, but no evidence could be found for a sex difference with respect to these connections.

### Efferent projections from anterior thalamic nuclei to the cingulate cortex

#### Anteromedial thalamic nucleus

**Figure 8.**
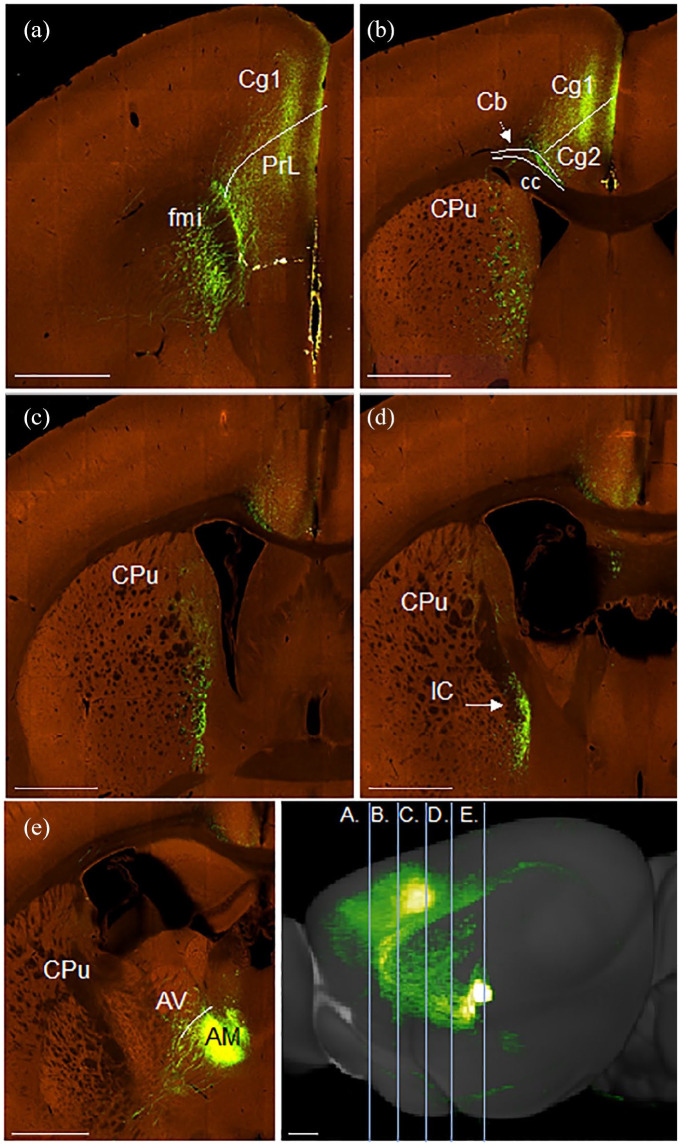
Photomicrographs showing the trajectory of fibres from the anteromedial thalamic nuclei to the cingulate cortex in the mouse. Coronal images (a–e, rostral to caudal) from an eGFP virus injection (case #573035760) in the Allen Mouse Brain Atlas (http://mouse.brain-map.org/). (a) Fibres wrapping around the genu in the forceps minor (fmi) of the corpus callosum. Terminal label in Cg1. (b) Fibres crossing the corpus callosum (cc) to enter the medial cingulum bundle (Cb). Termination in the anterior cingulate cortex (Cg1 and Cg2). (c) Fibres travelling rostralward through the medial caudoputamen (CPu). (d) Fibres leaving the thalamus anteriorly in the anterior limb of the internal capsule (IC). (e) Injection site centred in anteromedial thalamic nucleus (AM). (f) Sagittal reconstruction of anteromedial nucleus efferents with lines illustrating the anteroposterior level of images (a)–(e). AV: anteroventral thalamic nucleus; PrL: prelimbic cortex. Scale bars 930 µm. Images (a)–(f) (https://connectivity.brain-map.org/projection/experiment/573035760).

The course of these projections is illustrated in a representative case (#573035760) in which the eGFP virus injection was centred in the anteromedial nucleus, with very limited involvement of other thalamic nuclei (Figure 8(e)). It is evident that the fibres follow the same trajectory to the cortex as described in the rat. That is, fibres leave the thalamus rostrally in the anterior thalamic radiation (Figure 8(d)), then pass through the caudoputamen in fascicles (Figure 8(c)), with some fibres beginning to cross the corpus callosum from the level of the anterior commissure forward (Figure 8(b)). Other fibres wrap around the genu of the corpus callosum before turning caudally to join the medial part of the external medullary stratum of the cingulum bundle (Figure 8(a) and (b)). Cingulate cortex terminations also matched those seen in the rat, with label in layers 1 and 4–6 of Cg1 and Cg2 (Figure 8(e)) and layer 1 of Rgb.

#### Anteroventral thalamic nucleus

**Figure 9.**
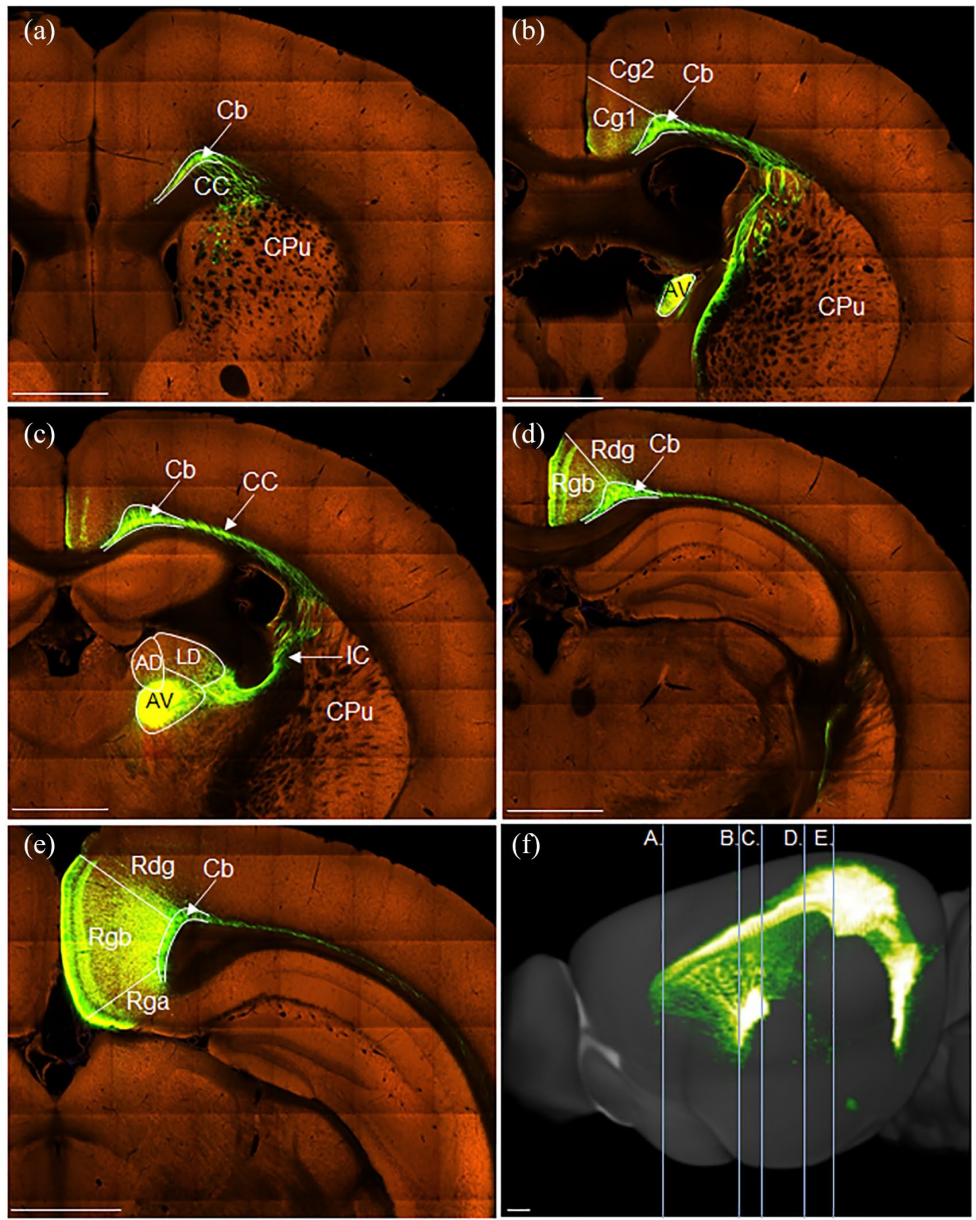
Photomicrographs showing the trajectory of fibres from the anteroventral thalamic nucleus to the cingulate and retrosplenial cortices of the mouse. Coronal images (a–e, rostral to caudal) from an eGFP virus injection (case #114427219) in the Allen Mouse Brain Atlas (http://mouse.brain-map.org/). (a) Fibres projecting in the rostral direction through the dorsal caudoputamen (Cpu), corpus callosum (CC) and cingulum bundle (Cb). (b) Projection of fibres from the anteroventral (AV) thalamic nucleus through the CPu to join the Cb. Some fibres change direction to reach the dorsal anterior cingulate cortex (Cg1) from a lateral direction. (c) Injection site centred in AV, with some spread to the adjacent anterodorsal (AD) thalamic nucleus. Many fibres leave the thalamus laterally, passing through the internal capsule (IC) and CPu to join the cingulum bundle (Cb). (d, e) Fibres move caudally through the Cb with terminal label seen mostly in the granular a and b retrosplenial cortex (Rga, Rgb), with some light terminal label in the dysgranular retrosplenial cortex (Rdg). (f) Sagittal reconstruction of anteroventral nucleus efferents with lines illustrating anteroposterior level of images (a)–(e). A ‘basket’ of fibres can be seen below the anterior cingulate cortex while the large majority of terminal label is in the retrosplenial cortex. Cg2: ventral anterior cingulate cortex; LD: laterodorsal thalamic nucleus. Scale bars 930 µm. Images (a)–(f) (https://connectivity.brain-map.org/projection/experiment/114427219).

Illustrations are provided for a representative case (#114427219) with a tracer injection principally located in the anteroventral nucleus (Figure 9). Additional attention was given to a transgenic case (#286553311) in which the injection appeared almost completely confined in the anteroventral nucleus (99%). In both cases, projections from the anteroventral nucleus follow the same trajectory as described in the rat. Some fibres emerge laterally from the injection site, taking a sharp dorsalward path through the corpus callosum and into the cingulum directly underneath the retrosplenial cortex (Figure 9(a)). Other fibres leave the thalamus anteriorly, before turning sharply dorsal through the caudoputamen to reach the cingulum (Figure 9(b)). Further fibres continue rostralward before entering the cingulum (Figure 9(c)), some reaching the genu of the corpus callosum (Figure 9(d)). A light projection terminated in anterior cingulate cortex, restricted to layer 1 of Cg2 (Figure 9(b)). In contrast to that seen after anteromedial injections, heavy termination was seen in retrosplenial cortex, particularly in layers 1–4 of Rgb (Figure 9(e)), with further light label in layer 1 of Rdg (Figure 9(e)).

The Allen Mouse Brain Atlas lacks a case in which the tracer injection is predominantly confined within the anterodorsal thalamic nucleus, so none is described.

### Efferent projections from cingulate cortex to the anterior thalamic nuclei

#### Anterior cingulate cortex

**Figure 10.**
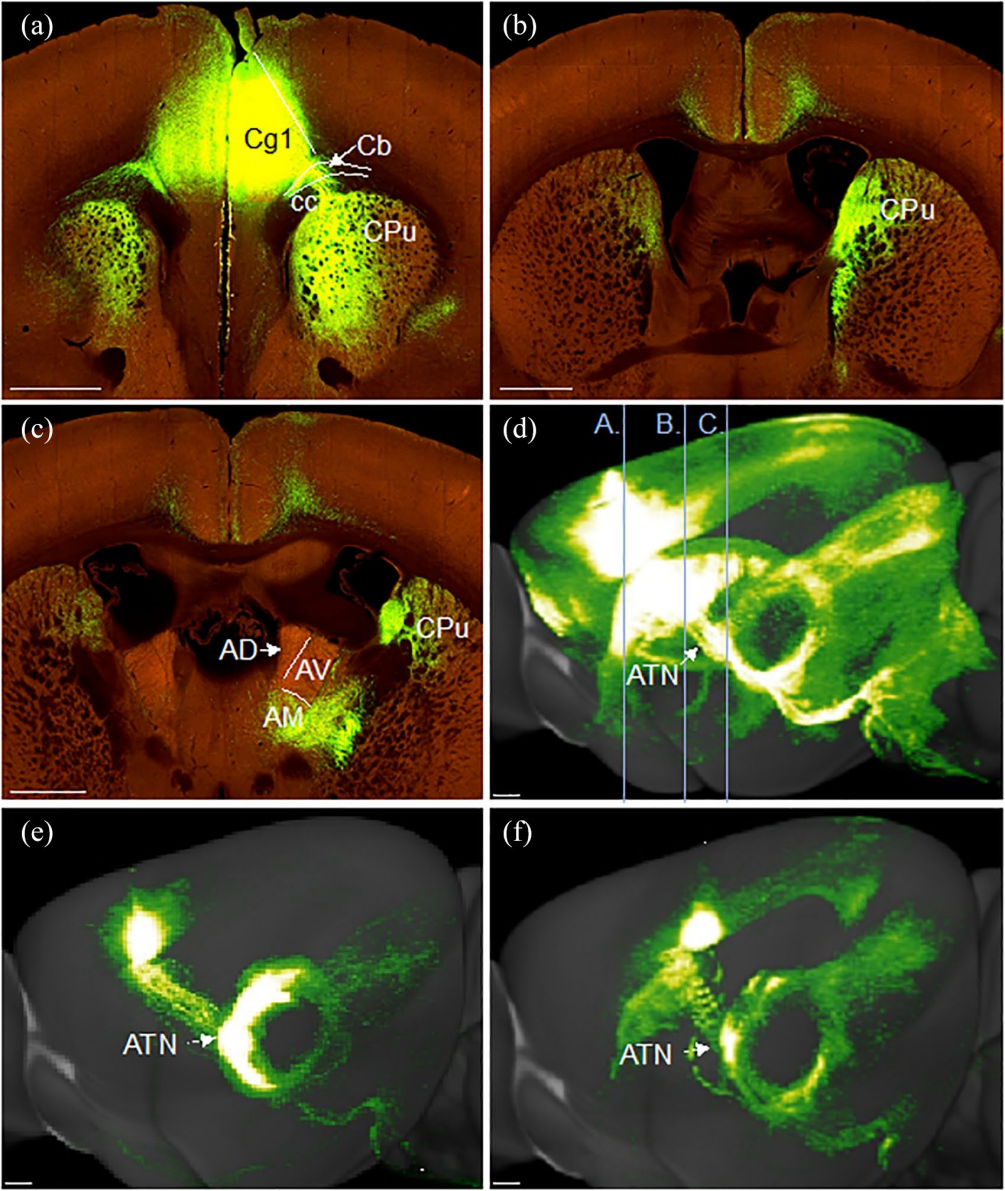
Photomicrographs showing the trajectory of fibres from the anterior cingulate cortex to the anterior thalamic nuclei (ATN) (http://mouse.brain-map.org/), (a–d, rostral to caudal, case #286482701; e, case #299829892; f, case #126190033). (a) Injection site centred in dorsal anterior cingulate cortex (Cg1) in #298482701. Fibres crossing caudally and ventrally through the cingulum bundle (Cb) and caudoputamen (CPu). (b) Fibres passing caudally through the medial CPu. (c) Fibres entering the thalamus to principally terminate in the anteromedial thalamic nucleus (AM). (d) Sagittal reconstruction of anterior cingulate cortex efferents with vertical lines illustrating anteroposterior level of images (a)–(c). (e, f) Sagittal reconstructions of two other anterior cingulate eGFP virus injections (e, #299829892; f, #126190033), illustrating different global projection patterns, but comparable projection routes to the anterior thalamic nuclei. AV: anteroventral thalamic nucleus; cc: corpus callosum. Scale bars 930 µm. Images in order of appearance: a–d (https://connectivity.brain-map.org/projection/experiment/286482701); e (https://connectivity.brain-map.org/projection/experiment/299829892); and f (https://connectivity.brain-map.org/projection/experiment/126190033).

Three cases were selected as representative of eGFP injections in the anterior cingulate cortex (Table 2). The injections varied in size and were located at different sites along the anteroposterior length of the region. Consequently, overall patterns of projections varied between cases, as evidenced by sagittal reconstructions (Figures 10(d)–(f)). Critically, however, the trajectories of fibres from each injection site to the anterior thalamic nuclei were comparable (Figures 10(d)–(f)) and matched descriptions in the rat.

Namely, fibres enter the internal stratum of the cingulum bundle at the level of the injection (Figure 10(a), case #286482701), before crossing through the corpus callosum and caudoputamen towards the anterior thalamic nuclei (Figure 10(b)). Here, the preponderance of terminal label was observed in the anteromedial nucleus (Figure 10(b)), with a light projection reaching the dorsomedial part of the anteroventral nucleus (AVDM). There was an absence of terminal label in the anterodorsal nucleus (Figure 10(c)). As can be seen from Figures 10(d)–(f), these cortico-thalamic projections typically took a direct route.

##### Retrosplenial cortex

**Figure 11.**
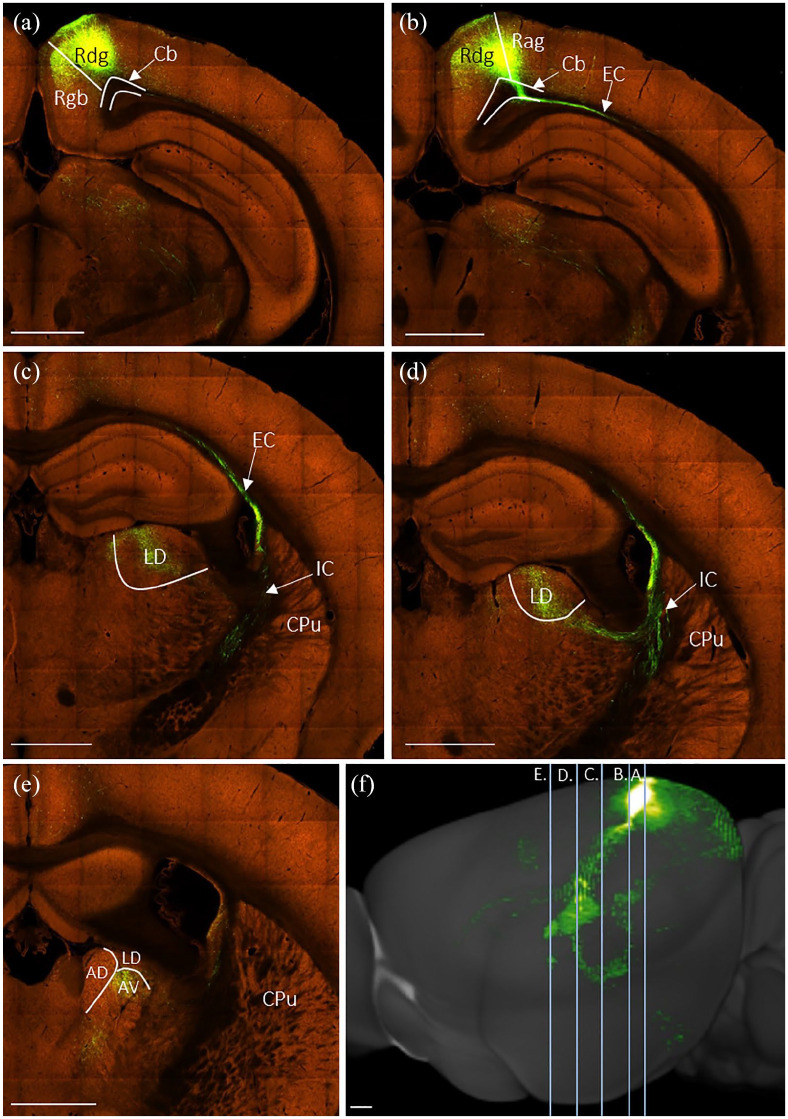
Photomicrographs showing the trajectory of fibres from retrosplenial cortex to the laterodorsal (LD) thalamic nucleus and the anterior thalamic nuclei in the mouse. Coronal images (a–e, caudal to rostral) from an eGFP virus injection (case #112424813) in the Allen Mouse Brain Atlas (http://mouse.brain-map.org/). (a) Injection site centred in dysgranular retrosplenial cortex (Rdg). (b) Fibres crossing the cingulum bundle (Cb) and entering the callosum and external capsule (EC). (c) Fibres passing from the external capsule into the internal capsule (IC) with some light termination in LD. (d) Fibres continue further through the IC with some termination in LD. (e) Termination in the anteroventral (AV) thalamic nucleus, with lighter termination in the anterodorsal (AD) and anteromedial (AM) thalamic nuclei. (f) Sagittal reconstruction of retrosplenial cortex efferents with lines illustrating anteroposterior level of images (a)–(e). The reconstruction shows how some retrosplenial fibres briefly join the cingulum heading rostral before turning lateral to cross the internal capsule and enter the thalamus on its dorsal lateral margin. CPu: caudoputamen; Rag: retrosplenial cortex, agranular; Rgb: retrosplenial cortex, granular b. Scale bars 930 µm. Images in order of appearance: a–f (https://connectivity.brain-map.org/projection/experiment/112424813).

For the three selected cases (Table 2), the eGFP injections were located along the anteroposterior length of the cortical region and together encompassed granular and dysgranular subdivisions. Typically, injections in retrosplenial cortex resulted in labelled fibres following a similar route to the thalamus as described in the rat (Figure 11, case # 112424813). Fibres entered the internal stratum of the cingulum bundle, before passing ventrally and rostrally in the external capsule (Figure 11(b)). Fibres then skirted the lateral ventricle in the internal capsule (Figure 11(c)), before turning medialward to enter the thalamus (Figure 11(d)). In this case, the heaviest terminal label was observed in laterodorsal (Figure 11(d)) thalamic nuclei. A lighter projection reached the anteroventral, anterodorsal and anteromedial thalamic nuclei (Figure 11(e)).

**Figure 12.**
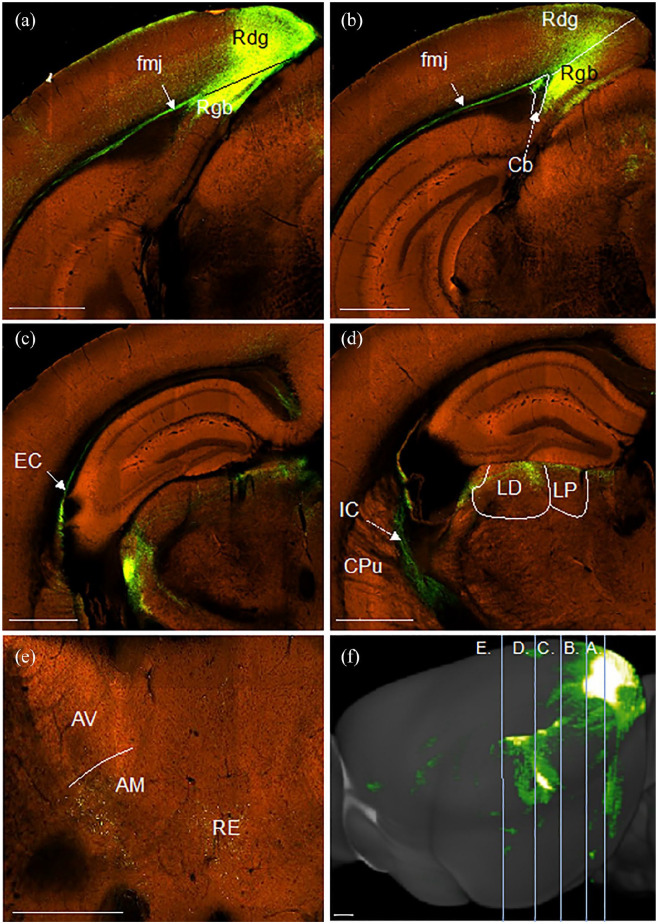
Photomicrographs showing the trajectory of fibres from caudal retrosplenial cortex to the thalamus in the mouse. Coronal images (a–e, caudal to rostral) from an eGFP virus injection (case #482581199) in the Allen Mouse Brain Atlas (http://mouse.brain-map.org/). (a) Injection site involving granular (Rgb) and dysgranular (Rdg) retrosplenial cortex. (b) Fibres running rostrally and ventrally in the cingulum bundle (Cb) and forceps major of the corpus callosum (fmj). (c) Fibres passing through the external capsule (EC). (d) Fibres crossing the caudoputamen (CPu) towards the internal capsule (IC), with termination visible in laterodorsal (LD) and lateral posterior (LP) thalamic nuclei. (e) Light terminal label in the anteromedial (AM) thalamic nucleus, as well as nucleus reuniens (RE). (f) Sagittal reconstruction of retrosplenial cortex efferents with lines illustrating anteroposterior level of images (a)–(e). This reconstruction shows how these cortico-thalamic projections largely avoid the cingulum bundle. AV: anteroventral thalamic nucleus; Rgb: granular retrosplenial cortex area b. Scale bars 930 µm. Images in order of appearance: a–f (https://connectivity.brain-map.org/projection/experiment/482581199).

It was, however, noted that some efferents from the more caudal aspects of retrosplenial cortex (granular and dysgranular) followed a slightly different route to the thalamus from that previously described (case #482581199, Figure 12), being more reliant on the external capsule. In this case, fibres crossed through the cingulum bundle to enter the forceps major of the corpus callosum (Figure 12(a) and (b)) at the level of the injection site. Fibres then continued ventralward and rostralward in the external capsule (Figure 12(c)), briefly joining the internal capsule before turning medialward to enter the thalamus. Here, heavy terminal label was observed in the laterodorsal and lateral posterior thalamic nuclei (Figure 12(d)), with a light projection reaching the anteromedial thalamic nucleus (Figure 12(e)).

## Discussion

The projections from the anterior thalamic nuclei to the cingulate cortices form a key stage in the circuit of Papez (1937), which has been classically associated with emotion (Dalgleish, 2004) and, more recently, with a wide range of cognitive abilities (Bubb et al., 2018). Despite anterior thalamic projections providing a key link in Papez circuit, there remains uncertainty over the course of the fibres (Weininger et al., 2019). Based on lesion-degeneration methods in rats, Domesick (1970) concluded that projections from the anterior thalamic nuclei to the anterior cingulate and retrosplenial cortices not only join the cingulum bundle but, in fact, comprise a major part of the pathway in this species. Domesick (1969) also reported that the return cortical projections take a more direct route to the anterior thalamus that only briefly involves the cingulum. Using a variety of axonal tracing methods, the present study both confirmed and extended Domesick’s original observations. The extension included the addition of data from the Allen Mouse Brain Atlas (http://mouse.brain-map.org/). While the latter includes many genetically modified mice, careful comparisons were repeatedly made with wild-type cases, but no evidence could be found of differences in the target projections.

### Anterior thalamic nuclei to the cingulate and retrosplenial cortices

**Figure 13.**
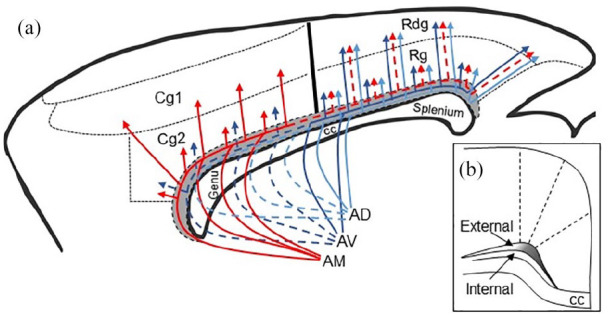
Sagittal schematic of the rat brain showing the routes taken by fibres from the anterior thalamic nuclei to the cingulate cortex. (a) The colours distinguish pathways from different nuclei. Dotted lines indicate lighter projections. The data are taken from the present study as well as prior publications cited in the text. The trajectories for AD efferents are inferred by the data in this study. Fibre pathways in the mouse match those of the rat. (b) Coronal schematic showing anterior thalamic efferents in the external medullary stratum of the cingulum. Shading represents distribution of fibres. AD: anterodorsal thalamic nucleus; AM: anteromedial thalamic nucleus; AV: anteroventral thalamic nucleus; Cb: cingulum bundle; cc: corpus callosum; Cg1: dorsal anterior cingulate cortex; Cg2: ventral anterior cingulate cortex; Rdg: retrosplenial dysgranular cortex; Rg: retrosplenial granular cortex.

In keeping with the differential distribution of projections from the anteromedial, anteroventral and anterodorsal nuclei to the cingulate cortices (Bubb et al., 2017; Shibata, 1993b; Sripanidkulchai and Wyss, 1986; Van Groen et al., 1999), there were clear differences in how axons from these nuclei often reach the cingulum bundle. Dense projections from the rat anteromedial nucleus leave the thalamus rostrally in the anterior thalamic radiation in fascicles that pass through the caudoputamen, often within the anterior limb of the internal capsule. Fibres then diverge, some continuing rostrally to course around the genu, where they join the cingulum bundle, others turn upwards to pierce through the corpus callosum and join the cingulum at multiple entry points. Some of the most rostral fibres reach medial prefrontal targets, including the pregenual dorsal anterior cingulate cortex (Cg1). Other fibres join the caudalward course of the cingulum bundle, where they aggregate in medial parts of the external medullary stratum (Figure 13(b)) before principally terminating in the anterior cingulate cortices. This rostral pattern of anteromedial projections reflects the close affinity of this nucleus with frontal cortical regions (Shibata and Naito, 2005; Wright et al., 2013). At the same time, only light anteromedial nucleus projections to the rostral retrosplenial cortex (Rgb) could be detected, although other research indicates additional, light projections to Rga (Van Groen et al., 1999) and Rdg (Shibata, 1993b) (Figure 13(a)).

There are similarities with projections from nucleus reuniens, which also pass around the genu to join the medial parts of the cingulum bundle (Wouterlood et al., 1990). While some of these projections terminate in the anterior cingulate and retrosplenial cortices, many continue caudally in the bundle beyond the retrosplenial cortex to terminate in hippocampal CA1, the dorsal subiculum, presubiculum and parasubiculum (Wouterlood et al., 1990).

Many of the projections from the anteroventral thalamic nucleus follow the same rostral route to the cortex as described for the anteromedial nucleus. Other fascicles, however, extend less far rostral in the caudoputamen before making a sharp upwards turn to cross into the cingulum bundle. Consequently, anteroventral efferents have more caudal entry points to the bundle than the projections from the anteromedial nucleus (Figure 13(a)). Meanwhile, a further set of anteroventral fibres leaves the thalamus laterally and enters the cingulum directly under the retrosplenial cortex (Figure 13(a)). This dual set of routes means that in both rats and mice the anteroventral nucleus has denser terminations in the retrosplenial cortex (Rgb and Rdg) than in Cg2 (see also Shibata, 1993b; Van Groen and Wyss, 1995). Although seemingly not previously reported in the rat, the lateral route from the anterior thalamic nuclei has been described in the monkey (Mufson and Pandya, 1984). Finally, within the cingulum bundle, there was a tendency for the anteroventral fibres to occupy a more central aspect of the external medullary stratum than fibres from the anteromedial nucleus.

Isolating the projections from the anterodorsal thalamic nucleus proved more challenging because of its size and location. Previous research has, however, shown that the anterodorsal nucleus projects to Rga and Rgb (Van Groen and Wyss, 1990, 1995, 2003), while our placement of retrograde tracers within different levels of the cingulum bundle provided supporting information. We found that few anterodorsal efferents join the rostral cingulum bundle, that is, at the level of the anterior cingulate cortex, consistent with a light projection to more caudal parts of this cortical area (Horikawa et al., 1988; Shibata and Naito, 2005). Rather, most anterodorsal nucleus efferents to the cortex join the cingulum bundle close to the level of the nucleus, that is, they leave the thalamus laterally and posteriorly to take the same lateral route as seen for a subpopulation of anteroventral efferents. This similarity in fibre trajectories reflects the close affinity of both the anteroventral and anterodorsal nuclei with more posterior cortical regions, such as the retrosplenial and parahippocampal cortices (Horikawa et al., 1988; Sripanidkulchai and Wyss, 1986; Van Groen and Wyss, 1995; Wright et al., 2013).

There are both similarities and differences in the rodent and monkey pathways. While in rats and mice the efferent anterior thalamic fibres occupy the external medullary stratum, in macaque monkeys these efferents are seen in the most ventral (internal) sector of the bundle (Mufson and Pandya, 1984). Unfortunately, studies of macaque monkeys have often not been able to separate the efferents from individual anterior thalamic nuclei, and so the described routes often appear to be an amalgam of those from the anteromedial and anteroventral nuclei, as well as the laterodorsal nucleus (Heilbronner and Haber, 2014; Mufson and Pandya, 1984). One route from the primate anterior thalamic nuclei, as in rodents, runs rostrally between the caudate nucleus and putamen, joining the anterior limb of the internal capsule, where some fibres continue before turning dorsal and then medial to reach the bundle near the genu, while other fibres turn dorsally in the internal capsule itself to head towards the fundus of the arcuate sulcus before turning medially to join the cingulum bundle (Mufson and Pandya, 1984). A second route leaves the anterior thalamus both laterally and posteriorly across the dorsal limit of the thalamus, before turning below and around the stria terminalis and caudate, then heading dorsally to cross the corpus callosum and join the cingulum bundle from its lateral aspect (Heilbronner and Haber, 2014; Mufson and Pandya, 1984). In the present study, this second route was also observed in the rodent brain but seemed restricted to anteroventral and anterodorsal nuclei efferents. An apparent difference is that in monkeys some fibres in this lateral route head rostral on joining the cingulum bundle, in addition to those that turn in a caudal direction (Heilbronner and Haber, 2014).

### Cingulate and retrosplenial cortices to the anterior thalamic nuclei

**Figure 14.**
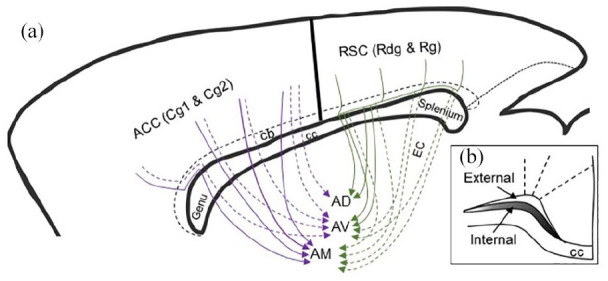
Sagittal schematic of the rat brain showing the routes taken by fibres from the cingulate cortex to the anterior thalamic nuclei. (a) The colours distinguish pathways from different cortical regions. Note that all projections from the anterior cingulate cortex are from both Cg1 and Cg2. Projections from retrosplenial cortex are from both its granular and dysgranular divisions. Dotted lines indicate lighter projections. The data are taken from the present study as well as prior publications cited in the text. Fibre pathways in the mouse match those of the rat. (b) Coronal schematic showing cingulate cortex efferents in the internal medullary stratum of the cingulum. Shading represents the distribution of fibres. Some of the most caudal retrosplenial projections involve the external capsule (EC). ACC: anterior cingulate cortex, dorsal (Cg1) and ventral (Cg2) subdivisions; AD: anterodorsal thalamic nucleus; AM: anteromedial thalamic nucleus; AV: anteroventral thalamic nucleus; cb: cingulum bundle; cc: corpus callosum; EC: external capsule; RSC: retrosplenial cortex, dysgranular (Rdg) and granular (Rg) subdivisions.

Analyses of the rat and mouse brain revealed comparable fibre trajectories from the anterior cingulate cortices to the anterior thalamic nuclei (Figure 14). Consistent with previous descriptions (Domesick, 1969), efferents from more caudal parts of the anterior cingulate cortex, that is, over the body of the corpus callosum, do not become enclosed in the cingulum but pierce through the white matter from their point of origin to reach the thalamus. Meanwhile, some projections from pregenual Cg1, which pass caudalward through the internal stratum of the cingulum (Figure 14) until over the body of the corpus callosum, only then turn ventral to follow the previously described direct route (Figure 14). The projections from the anterior cingulate cortex then terminate heavily in the anteromedial nucleus (Shibata and Naito, 2005; Wright et al., 2013), with a lighter projection to the anteroventral nucleus, largely restricted to its dorsomedial subregion (AVDM) (see also Shibata and Naito, 2005).

Meanwhile, efferents from the retrosplenial cortex to the anterior thalamic nuclei join the internal stratum of the cingulum bundle for a short length (Figure 14, see also Domesick, 1969), before crossing ventrally through the white matter to join the dorsal part of the internal capsule. These fibres then turn medial under the stria terminalis to reach the anterior thalamic nuclei, as well as the lateral dorsal thalamic nucleus. However, in the mouse, some thalamic afferents from the caudal retrosplenial cortex were identified that followed an additional trajectory. These fibres entered the forceps major of the corpus callosum from their point of origin to then initially continue ventrally in the external capsule before turning over 90° to cross the internal capsule and enter the dorsal margin of the thalamus. The majority of these caudal projections terminated in the laterodorsal and lateral posterior thalamic nuclei, but light projections reached the anterior thalamic nuclei. In line with previous descriptions (Mathiasen et al., 2017; Shibata, 1998; Van Groen and Wyss, 2003), efferents from the rat Rgb terminated in the anteroventral thalamic nucleus, with lighter termination in the anterodorsal nucleus. A light projection from Rdg terminated in the anteromedial nucleus (Shibata, 1998; Van Groen and Wyss, 1992).

Again, comparisons can be made with the corresponding cortico-thalamic projections in the nonhuman primate (Aggleton et al., 2014; Baleydier and Mauguiere, 1980; Mufson and Pandya, 1984). Tracing studies in macaque monkeys show that, as in the rat, these projections take a relatively direct route to the anterior thalamic nuclei and laterodorsal nucleus (Mufson and Pandya, 1984). Projections from anterior cingulate area 24 pass through the caudate nucleus into the anterior limb of the internal capsule to reach the thalamus. Meanwhile, projections from the more caudally placed posterior cingulate area 23 cut ventral and lateral across the corpus callosum to enter the internal capsule before turning medial to reach the thalamus (Mufson and Pandya, 1984).

### Similarities and differences: rats and mice

It was repeatedly found that both cortico-thalamic and thalamo-cortical connections took extremely similar courses in the rat and mouse brains. Consequently, the use of two, complementary data sets both increased the overall strength of the analyses and helped to compensate for those instances where specific injection targets were either lacking or insufficiently precise in one species. One example concerns the potential involvement of the reticular nucleus in the anterior thalamic injections, which could be discounted in the mouse data. Furthermore, the mouse data helped to show the lack of a sex difference in these connections.

### Implications

The increasing use of both structural and diffusion-based imaging to examine the rodent cingulum bundle (e.g. Figini et al., 2015; Paxinos et al., 2015) adds to the value of appreciating the changing composition of this complex pathway. Other implications relate to the outcome of conventional lesions of the cingulum bundle. Such surgeries have, for example, been made in rats to test their impact on cognition, with most studies focusing on spatial learning. Lesions largely restricted to the cingulum bundle typically produce only mild spatial deficits (Neave et al.,1996, 1997; Warburton et al., 1998), while related studies have found little evidence that cingulum bundle lesions add to the consequences of extensive retrosplenial damage (Harker and Whishaw, 2002, 2004)

The present study highlights how discrete cingulum bundle lesions will often only produce an incomplete disconnection of anterior thalamic–cortical interactions. First, many cortico-thalamic projections would be spared as a considerable number pass almost immediately through the bundle, that is, involve a limited anteroposterior extent of the bundle. Consequently, the most vulnerable fibres are those that run for some length along the bundle, for example, those from the anteromedial nucleus to the caudal half of the anterior cingulate cortex (Figure 13), as well as those from the anteroventral and anterodorsal nuclei to the retrosplenial cortex that do not use the direct lateral route (Figure 13). (Meanwhile those efferents that use the lateral route may well be largely spared.) These same surgeries might also be expected to disrupt the direct projections from the anterior thalamic nuclei to the hippocampal formation and parahippocampal region (Shibata, 1993a; Van Groen and Wyss, 1995), but many of these projections first join the more posterior parts of the cingulum. Furthermore, the cingulum bundle splays out dorsally above the splenium, making it a more diffuse target (Figure 5). This array of reasons may explain why the most severe spatial deficits followed tract lesions that extended dorsally above the caudal part of the main bundle (Aggleton et al., 1995). Finally, such surgeries will also disconnect projections from nucleus reuniens to the hippocampal region (Wouterlood et al., 1990), presumably contributing to any spatial deficits.

A related issue involves the fibre fascicles that pass though the caudoputamen, rostral to the anterior thalamic nuclei, en route to the cingulate cortices. Researchers interpreting the effects of conventional dorsal striatal lesions (e.g. Broadbent et al., 2007; Compton, 2004; Devan and White, 1999; Miyoshi et al., 2012; Thullier et al., 1996) have to consider the additional impact of anterior thalamic-cingulate cortex disconnection. Furthermore, mediodorsal thalamic nucleus projections to the frontal cortices also cross the striatum (Krettek and Price, 1977), as do nucleus reuniens – frontal connections (Wouterlood et al., 1990), adding to the complexity of interpreting such studies.

There is growing appreciation of the many functions that connections within the cingulum bundle serve. In addition to emotion and memory, the human cingulum bundle has been implicated in executive function (Bettcher et al., 2016) and pain (Corkin and Hebben, 1981), alongside a range of clinical conditions, including schizophrenia (Shepherd et al., 2012), depression (Bracht et al., 2015), post-traumatic stress disorder (Daniels et al., 2013), obsessive compulsive disorder (Radua et al., 2014), autism spectrum disorder (Travers et al., 2012), mild cognitive impairment (Metzler-Baddeley et al., 2012) and Alzheimer’s disease (Choo et al., 2010). This multiplicity of functions, allied to the complexity of the bundle (Heilbronner and Haber, 2014), helps to highlight the value of understanding better its component connections. This benefit extends to the rodent cingulum bundle, as rats and mice are increasingly used to model many of the disorders associated with cingulum dysfunction.
